# COS: A new MeSH term embedding incorporating corpus, ontology, and semantic predications

**DOI:** 10.1371/journal.pone.0251094

**Published:** 2021-05-04

**Authors:** Juncheng Ding, Wei Jin

**Affiliations:** Department of Computer Science and Engineering, University of North Texas, Denton, Texas, United States of America; University of Illinois-Chicago, UNITED STATES

## Abstract

The embedding of Medical Subject Headings (MeSH) terms has become a foundation for many downstream bioinformatics tasks. Recent studies employ different data sources, such as the corpus (in which each document is indexed by a set of MeSH terms), the MeSH term ontology, and the semantic predications between MeSH terms (extracted by SemMedDB), to learn their embeddings. While these data sources contribute to learning the MeSH term embeddings, current approaches fail to incorporate all of them in the learning process. The challenge is that the structured relationships between MeSH terms are different across the data sources, and there is no approach to fusing such complex data into the MeSH term embedding learning. In this paper, we study the problem of incorporating corpus, ontology, and semantic predications to learn the embeddings of MeSH terms. We propose a novel framework, Corpus, Ontology, and Semantic predications-based MeSH term embedding (COS), to generate high-quality MeSH term embeddings. COS converts the corpus, ontology, and semantic predications into MeSH term sequences, merges these sequences, and learns MeSH term embeddings using the sequences. Extensive experiments on different datasets show that COS outperforms various baseline embeddings and traditional non-embedding-based baselines.

## Introduction

Neural-based approaches have shown great success in bioinformatics applications, such as drug re-purposing and Literature-Based Discovery (LBD) [1–3]. The majority of such approaches take the terms’ distributed representations (embeddings) as inputs, making learning the term embedding a fundamental task in bioinformatics research. For example, given that heart disease is a type of cardiovascular disease, and fish oil can relieve heart disease, good embeddings of such terms can help indicate that fish oil may relieve other cardiovascular diseases and advance the biomedical research. Medical Subject Heading (MeSH) is a vocabulary of biomedical terms developed and maintained by domain experts and is useful in most bioinformatics applications [4]. Therefore, learning MeSH term embeddings has become an essential task and received considerable attention recently.

A primary line of MeSH term embedding learning uses the PubMed corpus as the data source. Since PubMed summarizes each document (publication) with a set of MeSH terms to describe its content, this line of research treats each MeSH term set as a document in the PubMed corpus to learn the MeSH term embeddings. In this line, [5–7] employ word embedding-based techniques [8, 9] to learn the MeSH term embeddings from the PubMed corpus. [10] also incorporates the ontology information in the embedding learning using word embedding-based techniques. These studies show that embedding-based approaches can improve downstream tasks’ performance over traditional non-embedding-based approaches. They also reveal that the PubMed corpus is useful to learn the MeSH term embeddings.

Another line of MeSH term embedding learning employs the semantic predications, extracted by SemMedDB, between MeSH terms to learn their embeddings [3, 11]. This line uses different knowledge graph embedding techniques such as TransE [12] to learn the embeddings of the MeSH terms via their semantic predications. The approach shows that semantic predications can also contribute to high-quality MeSH term embeddings.

Knowing that the MeSH vocabulary itself is carefully designed, more recent studies use the MeSH term ontology as a directed acyclic graph (DAG) in learning their embeddings [2, 13]. These approaches learn to represent the MeSH terms using graph embedding techniques. Their work shows that using such ontology information can achieve effective embeddings as well.

To conclude, current studies on learning MeSH term embeddings use different data sources in the learning process. The data sources can be categorized into three types: 1) the PubMed corpus, each document contains a set of MeSH terms describing its content; 2) the MeSH term ontology in DAG structure that is defined and maintained by the National Library of Medicine (NLM); 3) semantic predications extracted by SemMedDB, i.e., subject-predicate-object triples in SemMedDB where the subject and object are biomedical terms and the predicate is a semantic relationship. These approaches have shown that high-quality MeSH embeddings can effectively improve the performance of many downstream tasks. Moreover, all three data sources have also been shown useful in learning the embeddings of the MeSH terms. Recent progress in natural language processing has revealed that incorporating multiple data sources can further improve the term embedding learning [14–16]. However, no approach in MeSH term embedding learning merges all the three data sources to achieve better-quality embeddings.

Thus, it is natural to ask “whether using all the three data sources altogether will help the MeSH term embedding learning” and “how to incorporate all these data sources”. Since the structured relationships between MeSH terms are different across the three data sources, the challenge of current approaches in addressing the problems is that there is no existing method to model the three complex and different data sources in one embedding learning framework.

**Fig 1:**
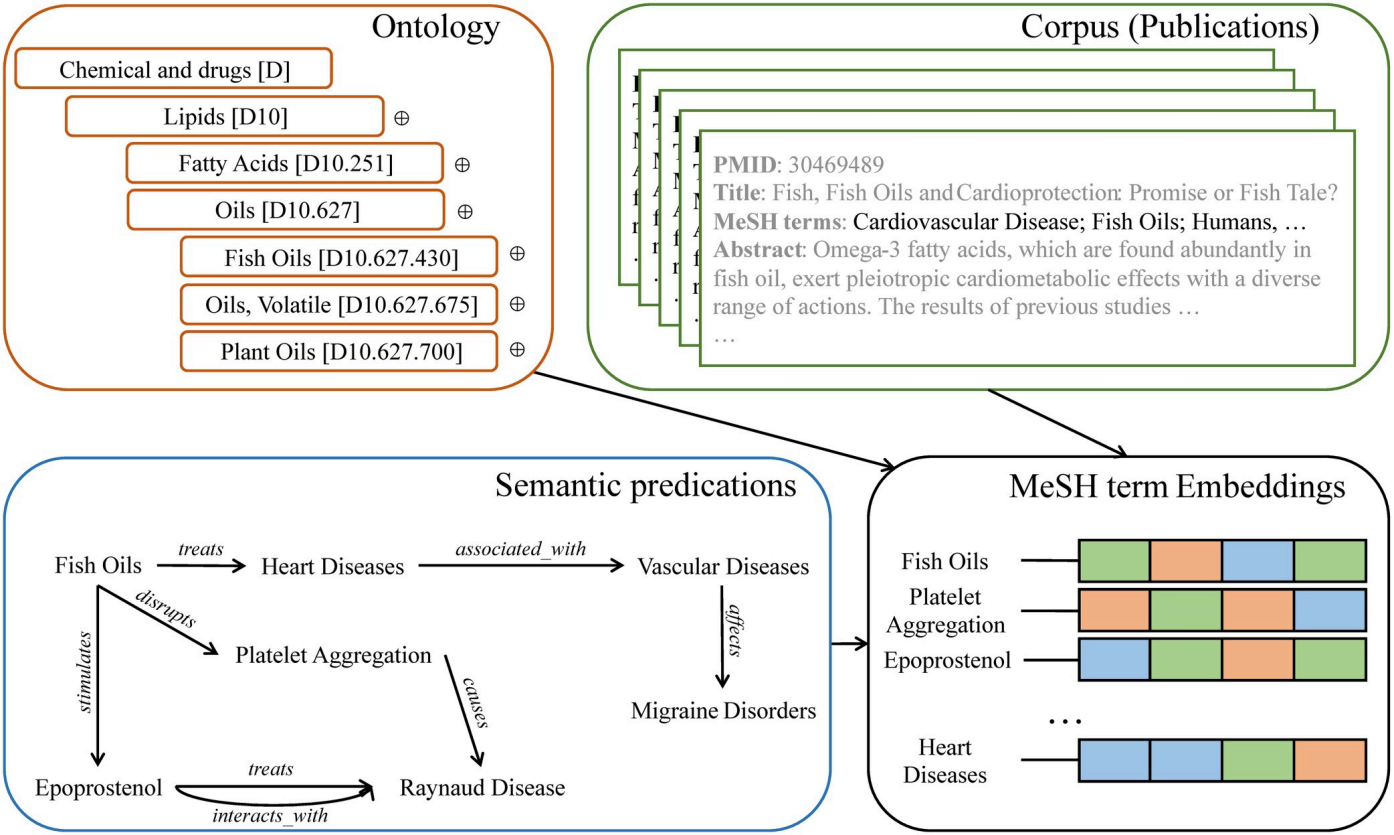
Problem definition. COS aims to learn MeSH term embeddings based on three data sources: corpus (the green block), ontology (the orange block), and semantic predications (the blue block). The structured relationships between MeSH terms are different across the data sources. The learned MeSH term embeddings should contain the information from all data sources.

**Fig 2:**
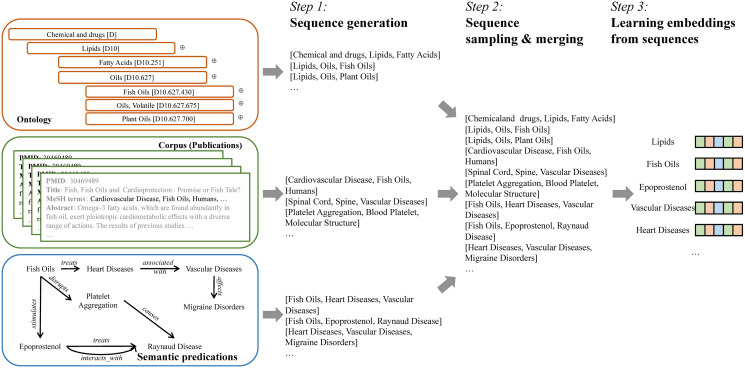
Our proposed solution. COS firstly generates MeSH term sequences from each data source. It then samples each group of generated sequences to the same number of sequences and merges them into one set of MeSH term sequences. Finally, COS learns the MeSH term embeddings based on the sequences set.

To address the challenge, we investigate learning MeSH term embeddings that incorporates the three data sources: corpus, ontology, and semantic predications as in Fig 1 in this paper. We propose *Corpus, Ontology, and semantic predications-based MeSH term embedding* (COS) to model all the three data sources in the embedding learning. COS uses all the three data sources in contrast with previous approaches that use only one or two of them as in Table 1. Recall that the ontology and semantic predications are graphs. In COS, we introduce an algorithm named *GraphSeqGen* (graph sequence generation) to generate MeSH term sequences from the two graphs. COS generates MeSH term sequences from the ontology and semantic predications using *GraphSeqGen* and samples MeSH term sequences from the PubMed corpus to learn the MeSH term embeddings. It then merges the generated sequences from the three data sources after a sampling process that up-samples each group of sequences into the same number of MeSH term sequences. Finally, COS optimizes the embeddings that best model those sequences. Fig 2 is the embedding learning process of COS.

**Table 1:** Summary of related work.

Methods	Corpus	Ontology	semantic predications
[6, 7]	✔		
[3, 11]			✔
[10, 17]	✔	✔	
[2, 13]		✔	
COS (proposed)	✔	✔	✔

In the experiments, we compare COS with MeSH term embeddings using different data sources on four datasets. The results show that the simple yet effective COS embedding outperforms both the baseline embeddings using each of the data source and the baseline embeddings that simply merge the data sources. COS also performs better than traditional non-embedding-based baselines in those tasks. While showing our proposed COS embedding’s effectiveness, the experiment results also justify our COS model design empirically. We will release our created datasets and source code upon acceptance.

We summarize our contribution as follows:

- We propose COS that incorporates the corpus, ontology, and semantic predications in MeSH term embedding learning, which is the first solution merging all the three data sources to the best of our knowledge.
- We compare COS with various baselines, showing its effectiveness. Moreover, the results also reveal that incorporating the three data sources improves the MeSH term embedding quality.
- We will make our pre-processed datasets and the COS source code publicly available.

## Methodology

This section first details the three data sources: corpus, ontology, and semantic predications. We present our proposed embedding learning framework COS after the data source introduction.

### Data sources

We describe the three data sources in this subsection. In the following description, we denote a MeSH term as *w*. Since the MeSH vocabulary is a controlled one, we have a fixed number of MeSH terms, and therefore denote the vocabulary as *V* containing all the MeSH terms *w*_*i*_, *i* = 1, …, *N*. Table 2 lists the important notation in this paper.

**Table 2:** Important notation.

Symbol	Definition
*w*_*i*_	a MeSH term in the MeSH vocabulary
*V* = {*w*_1_, …, *w*_*N*_}	the set containing all *N* MeSH terms
$d_{i}={\left\{w_{j}^{1},\ldots ,w_{j}^{n}\right\}}^{⊤}$	a document in PubMed which is a set containing a variable number of MeSH terms
$D=\{d_{1},\ldots ,d_{n}\}$	the PubMed corpus containing all publications **d**_*i*_
$E_{o}=\left\{e_{o}^{1},\ldots ,e_{o}^{n}\right\}$	the set containing all the edges in the ontology DAG
*G*_*o*_ = (*V*, *E*_*o*_)	the ontology data source as a directed graph
$E_{s}=\left\{e_{s}^{1},\ldots ,e_{s}^{n}\right\}$	the set of a specific type of semantic predications between MeSH terms in SemMedDB
*G*_*s*_ = (*V*, *E*_*s*_)	the semantic predications data source as a directed graph
*π*_*vx*_	the unnormalized transition probability between nodes *x* and *v* in a graph
*Z*	the normalizing constant
*p* and *q*	parameters of random walks interpolating between BFS and DFS
*α*_*pq*_(*t*, *x*)	the search bias parameter defined by *p* and *q* between nodes *t* and *x*
*w*_*vx*_	the weight of edge *e*_*vx*_ in a graph *G* = (*V*, *E*), equal to 1 in both *G*_*o*_ and *G*_*s*_
*r* and *l*	the number of walks per node and the walk length in random walks
$s_{i}={\left[w_{i}^{1},\ldots ,w_{i}^{n}\right]}^{⊤}$	a sequence of MeSH terms, generated from D, *E*_*o*_, or *E*_*s*_
$S_{c}=\left\{s_{c}^{1},\ldots ,s_{c}^{n}\right\}$	the set of sequences from the corpus sampled from D
$S_{o}=\left\{s_{o}^{1},\ldots ,s_{o}^{n}\right\}$	the set of sequences from the ontology graph *G*_*o*_
$S_{s}=\left\{s_{s}^{1},\ldots ,s_{s}^{n}\right\}$	the set of sequences from semantic predications graph *G*_*s*_
$S_{a}=\left\{s_{a}^{1},\ldots ,s_{a}^{n}\right\}$	the set of sequence after sampling and merging all sequences
*d* and *k*	the dimension of the embedding vector and the context window size in the optimization
$f:V\overset{}{\to }R^{d}$	the embedding mapping function, can be parameterized as a matrix of size |*V*| × *d*

#### Corpus

In PubMed, each document (publication) is assigned a set of MeSH terms summarizing its content [4]. Therefore, following [5–7, 10], we can use each set of MeSH terms to represent its respective document **d**. Each document **d** is a set of MeSH terms containing a variable number of *w*. The corpus D is a set containing all the documents **d**_*i*_ in PubMed. In this paper, we use the PubMed repository, last updated on March 23, 2020, as our corpus data source. The corpus includes 14,887,205 documents with more than one MeSH term and covers 28,358 MeSH terms.

#### Ontology

National Library of Medicine (NLM) has defined the ontology of all the MeSH terms as a DAG. In the ontology DAG, the node corresponding to *w*_*a*_ is pointed by the node corresponding to *w*_*b*_ if the MeSH term *w*_*a*_ is an instance of *w*_*b*_. In this regard, formally, we define the ontology of MeSH terms as a DAG *G*_*o*_ = (*V*, *E*_*o*_) [2]. In *G*_*o*_, the node set *V* contains all the MeSH terms *w*_*i*_, *i* = 1, …, *N* and is equivalent to the vocabulary *V* in the corpus data source. The edge set *E*_*o*_ contains all the edges in the ontology where an edge *e*_*o*_ from *w*_*a*_ to *w*_*b*_ represents that *w*_*a*_ is an instance of *w*_*b*_. In this paper, we use the ontology from NLM on September 22, 2019. The ontology covers 29,349 MeSH terms, and the number of edges is 39,784.

#### Semantic predications

SemMedDB has extracted many semantic predications between biomedical terms (including MeSH terms and non-MeSH terms) as subject-predicate-object triplets, where the subject and object are biomedical terms and the predicate is a specific semantic relationship. We use a specific type of semantic predications (i.e., predications containing a specific predicate *s*) to build a semantic predications graph *G*_*s*_ = (*V*, *E*_*s*_). In *G*_*s*_, the node set *V* is identical to that in *G*_*o*_ and contains only MeSH terms. The edge set *E*_*s*_ contains all the edges where an edge *e*_*s*_ between *w*_*a*_ and *w*_*b*_ means that *w*_*a*_ and *w*_*b*_ are related through the specific semantic predicate *s*. We create a graph *G*_*s*_ regarding a specific type of semantic predications by extracting predications that meet the three criteria from SemMedDB: 1) the subject is a MeSH term; 2) the object is a MeSH term; 3) the predicate is the *s* we specify.

We focus on four specific predicates (i.e., “treat”, “cause”, “interact”, and “affect”) in this paper, and conduct extensive experiments on the four respective generated graphs. We choose these four predicates as representatives of the four categories of predicates in SemMedDB: clinical medicine, substance interactions, genetic etiology of disease, and pharmacogenomics, respectively [18]. Moreover, they are also among the most frequent predicates in SemMedDB [19]. Table 3 presents the statistics of the four semantic predications graph.

**Table 3:** The four semantic predications datasets and their statistics.

Dataset	treat	interact	cause	affect
MeSH term count	9,277	8,336	10,393	12,020
Predications count	178,406	260,762	148,378	261,677

### Embedding learning framework

Our proposed feature learning framework has three steps as in Fig 2:

1. Transforming all the data sources into sets of sequences as $S_{c}\overset{}{\leftarrow }D$, $S_{o}\overset{}{\leftarrow }G_{o}$, and $S_{s}\overset{}{\leftarrow }G_{s}$. We transform data sources, in which the structured relationships between MeSH terms are different, into sequences to unify them into the same form. We can thus merge the sequences from the different data sources and use the merged sequences to learn the embeddings.
2. Sampling and merging all these sequences via $S_{a}=⋃\{\text{Sample}\left(S_{c},N\right),\text{Sample}\left(S_{o},N\right),\text{Sample}\left(S_{s},N\right)\}$, where $N=\text{max}(len\left(S_{c}\right),len\left(S_{o}\right),len\left(S_{s}\right))$. In the above equation, Sample (S,L) is the process of sampling S into an *L*-sized set $S^{\prime }$ with replacement. We introduce the up-sampling procedure before merging the sequences from different data sources to compensate for the unequal numbers of sequences from each source. Therefore, after up-sampling, the data sources will contribute equally to the embedding learning.
3. Learning the MeSH term embeddings from $S_{a}$. This step learns the MeSH term embeddings using the merged sequences based on stochastic gradient descent. Specifically, we learn an embedding mapping function *f*, which maps the MeSH terms into their respective embeddings, via $f=\text{StochasticGradientDescent}(k,d,S_{a})$, where *d* is the dimension of embedding vectors and *k* in the context window in the learning process as in Table 2.

We describe the three steps below.

#### Sequence generation

This step transforms the three data sources into three sets of sequences, i.e., step 1 in the embedding learning framework section. We sample a MeSH term sequence from each set of MeSH terms in the corpus data source via uniform random sampling following previous approaches [5–7, 10], and will focus on transforming the ontology graph *G*_*o*_ and the semantic predications graph *G*_*s*_ into MeSH term sequences. We argue that we can use the same algorithm to generate sequence sets from the two graphs because they are both DAGs. We propose a *GraphSeqGen* (Graph Sequences Generation) algorithm based on random walk [20, 21] to generate sequences, i.e., S=GraphSeqGen(G). The intuition behind *GraphSeqGen* is to generate MeSH term sequences via randomly walking in the MeSH term graph. The paths of the random walks are, therefore, the generated MeSH term sequences. Algorithm 1 presents the proposed *GraphSeqGen* algorithm.

**Algorithm 1 *GraphSeqGen***

**Input**: graph *G* = (*V*, *E*), return *p*, in-out q*, walks per term *r*, walk length *l*

**Output**: generated set of sequences S

 *π* = PreprocessModifiedWeights(*G*, *p*, *q*)

 *G*′ = (*V*, *E*, *π*)

 Initialize S to {};

 **for** *i* = 1 **to** *r* **do**

  **for all** terms *w* ∈ *V* **do**

   **s** = node2vecWalk(*G*′, *w*, *l*)**

   Add **s** to S

  **return** S

* The return parameter *p* and the in-out parameter *q* are two parameters deciding how to sample the next step during the random walks as in Eq 2.

** The algorithm *node2vecWalk* is the one in [21] that generates a sequence of length *l* starting from the term *w* in the graph with modified weights.

To ensure that the paths of random walks can capture the feature of a graph, *GraphSeqGen* adopts the algorithm in [21] that combines the depth-first sampling (DFS) and the breadth-first sampling (BFS). The algorithm walks in a graph via a designed transition probability matrix that interpolates both DFS and BFS. The transition probability from node *w*_*v*_ to *w*_*x*_ is *P*(*x*|*v*) defined in Eq 1.

$$\begin{array}{c} P\left(x|v\right)=\{\begin{array}{cc} \frac{\pi_{vx}}{Z}, & \text{if}\;(v,x)\in E\\ 0, & \text{otherwise} \end{array} \end{array}$$ (1)

where *Z* is a normalizing constant, and *π*_*vx*_ is defined in Eq 2. *d*_*tx*_ in Eq 2 is defined in the process of a two-node walk on the graph where walking two nodes will lead to a node that can be 0, 1, and 2 edges away from the original node (i.e. there exist multiple paths of different lengths from the start node to the end node). In the walking process, *d*_*tx*_ = 0 if we walk 0 edges away, *d*_*tx*_ = 1 if we walk 1 edge away, and *d*_*tx*_ = 2 if we walk 2 edges away.

We can assign different probabilities to different *d*_*tx*_ to control how to traversal the graph to generate sequences, i.e., whether to prefer DFS or BFS. This design of the probability, defined by *p* and *q*, ensures the generated MeSH term sequences can well capture the graph information [21].

$$\begin{array}{c} \pi_{vx}=\{\begin{array}{cc} \frac{1}{p}, & \text{if}\;d_{tx}=0\\ 1, & \text{if}\;d_{tx}=1\\ \frac{1}{q}, & \text{if}\;d_{tx}=2 \end{array} \end{array}$$ (2)

After generating the transition probability matrix *π*, we employ the *node*2*vecWalk* algorithm, as in [21], that generates sequences via “randomly walking” starting from each node. *r* defines the number of sequences for each node and *l* defines the length of paths (MeSH term sequences).

Using Algorithm 1 independently on the ontology graph *G*_*o*_ and the semantic predications graph *G*_*s*_, we can generate the two sets of sequences $S_{o}$ and $S_{s}$ from the two data sources respectively, via S=GraphSeqGen(G).

#### Sequence sampling and merging

We describe step 2 in this section. The MeSH term sequence sets $S_{c}$, $S_{o}$, and $S_{s}$ are different in their total numbers of sequences. The difference will lead to the data sources’ different contributions to the learned embeddings if we simply merge the three sets of sequences, which could impact the final embeddings’ quality. We propose a sampling algorithm to generate the same number of sequences from different data sources to address the above problem. Specifically, we find the number of sequences in the largest set and fix it as the number of sequences in each set (i.e., $\text{max}(\text{len}\left(S_{c}\right),\text{len}\left(S_{o}\right),\text{len}\left(S_{s}\right)$). Afterwards, we up-sample the two smaller sets to the fixed number of MeSH term sequences with replacement.

After sampling, we will get three (more specifically, two and the other one is original) up-sampled sets of sequences $S_{c}^{\prime }$, $S_{o}^{\prime }$, and $S_{s}^{\prime }$, respectively. We merge these three sets to generate the final sets of sequences $S_{a}$ for embedding learning, via $S_{a}=S_{c}^{\prime }⋃\;S_{o}^{\prime }⋃\;S_{s}^{\prime }$.

It is worth mentioning that we have also experimented with the learned embeddings 1) without the above algorithm up-sampling and 2) with down-sampling to the original number after up-sampling, i.e., we will use $S_{a}^{\prime }=\text{Sample}\left(S_{a},len\left(S_{c}\right)+len\left(S_{o}\right)+len\left(S_{s}\right)\right)$, where $S_{a}$ is the sequences set after up-sampling, to learn the MeSH term embeddings instead of $S_{a}$. The experiments show that it is this sampling algorithm, which ensures that the three data sources contribute equally to the embeddings, that improves the quality of the embeddings rather than the larger number of sequences introduced by up-sampling.

#### Learning embeddings from sequences

In the final step of our proposed framework, our goal is to learn $f:V\overset{}{\to }R^{d}$ that maps a MeSH term *w* into a *d*-dimensional real-valued vector (embedding) based on $S_{a}$. Specifically, we adopt the “skip-gram” model [9] to learn *f* using the merged MeSH term sequences $S_{a}$. The assumption is that we should be able to predict ∀*w*_*i*_ ∈ *N*(*w*) given *w* using *f*, where *N*(*w*) is the window of MeSH terms in a sequence centered on the MeSH term *w* (i.e., *w*_*i*_ is in *N*(*w*) if *w*_*i*_ is not *w* and *w*_*i*_ is in a *k*-sized window centered on *w* of a MeSH term sequence), based on the likelihood. Therefore, our problem is a maximum likelihood optimization one. The log-likelihood is $\sum_{w\in s}\sum_{w_{i}\in N(w)}\text{log}p(w_{i}|f(w))$ for a sequence $s\in S_{a}$ where the probability *p*() is defined as the softmax of the two embeddings’ dot product as in Eq 3. The objective function is the sum of the likelihoods of all sequences in $S_{a}$ as below:

$$\begin{array}{c} \begin{array}{c} \max_{f}\sum_{s\in S_{a}}\sum_{w\in s}\sum_{w_{i}\in N\left(w\right)}\log p(w_{i}|f\left(w\right)),\text{where}\\ p\left(w_{i}|f\left(w\right)\right)=\frac{\exp(f{\left(w_{i}\right)}^{⊤}f\left(w\right))}{\sum_{u\in V}\exp(f{\left(u\right)}^{⊤}f\left(w\right))} \end{array} \end{array}$$ (3)

In Eq 3, the term ∑_*u*∈*V*_ exp(*f*(*u*)^⊤^ *f*(*w*)) is impractical to compute for a large vocabulary. Therefore, we adopt the algorithm of negative sampling [9] to approximate it. The above terms thus become ∑_*u*∈*S*(*w*)_exp(*f*(*u*)^⊤^ *f*(*w*)), where *S*(*w*) includes several MeSH terms randomly drawn from a uniform distribution.

We learn *f* via optimizing Eq 3 using stochastic gradient ascent. After several iterations, we will get *f* that maps each of the *V* MeSH terms into a *d*-dimensional real-valued vector (embedding). The mapping function *f* is in the form of a lookup dictionary parameterized as a |*V*| × *d*-sized matrix.

To ensure consistency, we use the recommended parameters and fix them in our model and all comparatives in our experiments. The transition probability parameters *p* and *q* are 0.25 and 4. The number of walks *r* and the walk length *l* are 80 and 10. The dimension *d* of the embeddings is 128. The window size *k* is 5.

## Experiments

We describe our experiments and analyze the results in this section. Before presenting the results, we detail our experiment setting and baselines.

### Experimental setting

Most of the downstream bioinformatics tasks rely on the quality of the term embedding and the representation of links between terms [1]. As in [22], we can use the link (i.e., semantic relationship or edge in the semantic predications graph *G*_*s*_) prediction performance to evaluate both the term embedding and the edge (or semantic relationship) representation’s quality. Therefore, we will evaluate our MeSH term embeddings via edge prediction tasks on four different semantic predications graphs as in Table 3.

In our experiments, an edge in *G*_*s*_ is a semantic predication containing a specific predicate *s* between two MeSH terms, and an existing semantic predication in SemMedDB is a valid edge. The edge prediction task is a binary classification problem in which we need to classify whether a previously unseen edge is valid or invalid. We will describe our data preparation, edge representation learning, and edge prediction setting in the remaining of this subsection.

#### Data preparation

Each dataset includes many valid edges as in Table 3. To create our dataset for edge prediction, we split the valid edges by 50%, 25%, and 25% as positive samples for the training set, the validation set, and the testing set, respectively. We sample the same number of invalid edges (edges not present in SemMedDB) for each set as negative samples. We get three balanced sets of edges as our training dataset, validation dataset, and testing dataset via merging the respective positive and negative samples.

During the above splitting process, we maintain that the graph with only the training set’s valid edges have the same connectivity as the original graph (i.e., if there is a path between *w*_*a*_ and *w*_*b*_ in the original graph, there should be a path between *w*_*a*_ and *w*_*b*_ in the graph containing only valid edges in the training dataset), ensuring we can learn meaningful MeSH term embeddings using the graph containing only valid edges in the training dataset. As for the implementation, we guarantee that the valid edges in the training dataset contains all the bridging edges in the original graph.

#### Edge representation learning

This step learns the representation of edges between MeSH terms. We first generate the MeSH term embeddings as *f* using COS and different baselines. The second step generates the representation of edges between MeSH terms as $g:V\times V\overset{}{\to }R^{d}$ based on *f*(.). Following the previous work [2, 21, 22], we generate the edge representation via the MeSH term embedding and the average operator. Specifically, an edge representation between MeSH term *w*_*u*_ and *w*_*v*_ is a *d*-dimensional real-valued vector returned by *g*(*w*_*u*_, *w*_*v*_), which is defined as $g\left(w_{u},w_{v}\right)=\frac{f\left(w_{u}\right)+f\left(w_{v}\right)}{2}$.

Note that we use only the valid edges in the edge prediction training set to build our semantic predications graph *G*_*s*_ rather than all the valid edges, ensuring that the training process does not see the testing data, in the MeSH term embedding learning process. We use all the documents in the corpus and the whole ontology as D and *G*_*o*_ respectively.

#### Edge prediction setting

The previous two steps have created the training samples, the validation samples, the testing samples and the samples’ representations for the edge prediction task. We use the training samples to train our classifier, the validation samples to judge when to stop training (or whether the classifier is over-fitting the training samples), and the testing samples to evaluate the performance. Our classifier is a two-layer densely connected neural network with a hidden size of 256 and a ReLU activation function in each layer. The output layer is a softmax. The loss function is the cross-entropy loss. We train the model with a maximum of 2000 epochs and adopt an early stopping algorithm that stops training when the validation set’s loss does not decrease for ten epochs. We build our classifier and implement the training process on top of Keras.

We evaluate the performance of edge prediction using P (precision), R (recall), F1, MAP (mean average precision, the mean of averaged precision over all thresholds), AUROC (area under the receiver operating characteristics curve), and AUPRC (area under the precision-recall curve). Those scores can measure the quality of the MeSH term embeddings and the representation of edges between MeSH terms [22].

### Baselines

Our baseline approaches contain two groups: 1) the non-embedding-based edge prediction approaches; 2) the embedding-based edge prediction approaches.

Since our task is an edge prediction problem in graphs, we compare our approach with traditional non-embedding-based approaches designed for graphs to justify the advantage of embedding-based approaches. The non-embedding-based edge prediction approaches predict the edges using measurements based on graph connectivity. We include the four most recognized methods Jaccard, preferential attachment, Adamic-Adar, and common neighbors [23] in this group of baseline approaches.

To measure the quality of our proposed COS embeddings, we compare it with other embeddings in these edge prediction tasks. This group of experiments use different embeddings but follow the same procedure as in the experimental setting section. We compare with four groups of baseline embeddings: 1) the embedding from corpus D using word2vec [9]; 2) the embeddings from ontology graph *G*_*o*_ using five recognized graph embedding techniques DeepWalk [20], LINE [24], Node2vec [21], SDNE [25], and Struc2vec [25]; 3) the embeddings from semantic predications graph *G*_*s*_ using the same five graph embedding techniques; 4) the embeddings that merge respective embeddings from the three data sources via averaging them. Note that we only use the valid edges in the training set in creating *G*_*s*_ to prevent the training process from being exposed to the testing data.

### Experiment results

In this section, we compare our model with numerous baselines to show our proposed model’s effectiveness. We also present experiments justifying our COS model design.

#### Model comparisons

We compare COS with the baselines in this subsection. Note that the random initialization can impact the performance of embedding-based approaches. To mitigate the impact, we run each setting ten times in our embedding-based experiments and present the ten runs’ average scores. We have also conducted statistical significance tests between the best-performing approach and the rest approaches. Specifically, for any two different approaches, we have conducted a two-sided *t*-test of the ten runs’ scores by each approach.

We present our experiment results on the four datasets in Tables 4–7. The bold numbers indicate that the respective settings have achieved the best performance within the dataset. The numbers followed by a * sign indicate the respective settings have achieved statistically significant (*p*-value < 0.001) difference in performance from all the other baseline approaches.

**Table 4:** Edge prediction results for treat.

Method		P(%)	R(%)	F1(%)	MAP(%)	AUROC(%)	AUPRC(%)
Jaccard		49.83	49.83	33.26	50.00	49.83	25.00
preferential attachment		52.72	52.72	39.71	51.40	52.72	75.50
Adamic-Adar		48.86	48.86	33.04	49.90	48.86	29.56
common neighbors		44.58	44.58	33.58	48.87	44.58	36.41
corpus	word2vec	83.01	83.01	83.00	77.98	83.02	87.55
ontology	DeepWalk	74.62	74.62	74.59	68.17	74.62	81.02
	LINE	66.48	66.48	66.19	61.53	66.48	74.51
	Node2vec	82.44	82.44	82.43	76.45	82.44	86.75
	SDNE	50.76	50.76	36.19	50.47	50.76	67.17
	Struc2vec	66.85	66.85	66.72	61.24	66.85	75.42
semantic predications	DeepWalk	81.09	81.20	81.09	77.00	81.20	86.76
	LINE	83.13	83.23	83.13	79.27	83.23	88.26
	Node2vec	81.05	81.06	81.05	76.20	81.06	86.38
	SDNE	87.35	87.39	87.35	83.71	87.39	91.09
	Struc2vec	85.90	85.87	85.88	81.31	85.87	89.73
merged	DeepWalk	81.24	81.23	81.22	76.33	81.23	86.49
	LINE	83.36	83.38	83.36	78.85	83.38	88.10
	Node2Vec	84.83	84.80	84.81	80.15	84.80	89.00
	SDNE	88.35	88.35	88.34	84.59	88.35	91.65
	Struc2Vec	83.98	83.96	83.97	79.16	83.96	88.38
COS (proposed)		91.38*	91.38*	91.37*	88.30*	91.38*	93.78*

* denotes statistically significant difference (*p* < 0.001) compared to all the above baselines.

**Table 5:** Edge prediction results for interact.

Method		P(%)	R(%)	F1(%)	MAP(%)	AUROC(%)	AUPRC(%)
Jaccard		49.97	49.97	33.33	50.00	49.97	29.17
preferential attachment		52.44	52.44	38.91	51.25	52.44	75.51
Adamic-Adar		88.51	88.51	88.47	86.00	88.51	92.44
common neighbors		86.53	86.53	86.42	79.63	86.53	89.36
corpus	word2vec	85.65	85.65	85.64	80.65	85.65	89.30
ontology	DeepWalk	72.60	72.60	72.57	66.43	72.60	79.48
	LINE	69.55	69.55	69.31	64.36	69.55	77.09
	Node2vec	78.59	78.59	78.54	72.09	78.59	83.96
	SDNE	52.16	52.16	41.39	51.41	52.16	59.89
	Struc2vec	70.54	70.54	70.45	64.84	70.53	77.88
semantic predications	DeepWalk	84.55	84.59	84.55	80.45	84.59	89.07
	LINE	84.37	84.47	84.37	80.75	84.47	89.20
	Node2vec	82.94	82.93	82.92	78.00	82.93	87.63
	SDNE	87.24	87.28	87.24	83.51	87.28	90.97
	Struc2vec	86.04	86.02	86.02	81.50	86.02	89.83
merged	DeepWalk	84.69	84.67	84.67	79.91	84.67	88.85
	LINE	85.32	85.31	85.31	80.74	85.31	89.35
	Node2Vec	86.34	86.30	86.32	81.69	86.30	89.98
	SDNE	88.62	88.61	88.61	84.64	88.61	91.71
	Struc2Vec	85.42	85.40	85.40	80.74	85.40	89.37
COS (proposed)		90.13*	90.13*	90.13*	86.34*	90.13*	92.69*

* denotes statistically significant difference (*p* < 0.001) compared to all the above baselines.

**Table 6:** Edge prediction results for cause.

Method		P(%)	R(%)	F1(%)	MAP(%)	AUROC(%)	AUPRC(%)
Jaccard		49.96	49.96	33.32	50.00	49.96	25.00
preferential attachment		52.95	52.95	40.58	51.52	52.95	75.42
Adamic-Adar		69.68	69.68	66.88	69.10	69.68	83.68
common neighbors		79.56	79.56	79.45	74.95	79.56	85.33
corpus	word2vec	83.23	83.24	83.23	78.22	83.24	87.70
ontology	DeepWalk	75.29	75.29	75.27	68.94	75.29	81.50
	LINE	68.58	68.58	68.45	63.23	68.58	76.23
	Node2vec	81.64	81.64	81.60	75.15	81.64	86.10
	SDNE	50.80	50.80	36.32	50.50	50.80	64.63
	Struc2vec	69.27	69.27	68.97	64.07	69.27	77.01
semantic predications	DeepWalk	81.57	81.75	81.55	78.20	81.75	87.50
	LINE	82.67	82.74	82.65	78.54	82.74	87.86
	Node2vec	79.21	79.05	79.09	73.34	79.05	84.89
	SDNE	87.82	87.81	87.81	83.81	87.81	91.21
	Struc2vec	87.62	87.59	87.61	83.44	87.59	91.01
merged	DeepWalk	82.17	82.15	82.15	77.36	82.15	87.18
	LINE	84.06	84.05	84.05	79.48	84.05	88.54
	Node2Vec	84.62	84.62	84.61	80.18	84.62	88.97
	SDNE	88.44	88.44	88.43	84.79	88.44	91.76
	Struc2Vec	85.13	85.11	85.11	80.60	85.11	89.27
COS (proposed)		90.09*	90.09*	90.08*	86.55*	90.09*	92.79*

* denotes statistically significant difference (*p* < 0.001) compared to all the above baselines.

**Table 7:** Edge prediction results for affect.

Method		P(%)	R(%)	F1(%)	MAP(%)	AUROC(%)	AUPRC(%)
Jaccard		49.96	49.96	33.32	50.00	49.96	25.00
preferential attachment		53.13	53.13	40.52	51.62	53.13	75.61
Adamic-Adar		75.84	75.84	74.67	74.64	75.84	86.26
common neighbors		80.58	80.58	80.57	74.84	80.58	85.50
corpus	word2vec	83.96	83.97	83.96	79.05	83.97	88.24
ontology	DeepWalk	76.24	76.24	76.23	70.10	76.24	82.21
	LINE	71.08	71.08	70.90	65.79	71.08	78.33
	Node2vec	83.25	83.25	83.24	77.34	83.25	87.35
	SDNE	52.26	52.26	39.54	51.73	52.26	67.53
	Struc2vec	71.71	71.71	71.65	65.91	71.71	78.77
semantic predications	DeepWalk	76.24	76.24	76.23	70.10	76.24	82.21
	LINE	71.08	71.08	70.90	65.79	71.08	78.33
	Node2vec	83.25	83.25	83.24	77.34	83.25	87.35
	SDNE	52.26	52.26	39.54	51.73	52.26	67.53
	Struc2vec	71.71	71.71	71.65	65.91	71.71	78.77
merged	DeepWalk	83.64	83.61	83.61	78.89	83.61	88.20
	LINE	85.52	85.48	85.49	80.96	85.48	89.52
	Node2Vec	85.66	85.64	85.64	81.27	85.64	89.68
	SDNE	88.76	88.74	88.75	84.93	88.74	91.88
	Struc2Vec	86.05	86.00	86.03	81.46	86.00	89.85
COS (proposed)		91.82*	91.82*	91.82*	88.51*	91.82*	93.92*

* denotes statistically significant difference (*p* < 0.001) compared to all the above baselines.

From Tables 4–7, we can find that the embedding-based approaches generally perform better than the traditional non-embedding-based baselines in our biomedical edge prediction tasks. The observation empirically proves the necessity of using embeddings in biomedical edge prediction tasks. The results also show that the embedding-based approaches using semantic predications generally outperform the other two groups of embedding-based approaches using the the corpus and the ontology as data sources. Such results can be explained by the fact that the edge prediction is a semantic predications-based task and could benefit from using related data sources. Another finding is that the corpus-based approach performs better than the ontology-based approaches most of the time, which can be explained by that the large corpus contains richer latent semantic information than solely the ontology because of its huge volume. Moreover, we can see that the five different graph embedding approaches perform differently on the ontology and semantic predications data sources. The reason is that the structures of *G*_*o*_ and *G*_*s*_ are different and the embedding learning approaches perform differently on different-structured graphs. However, the performance of Node2vec is stable across all of the “ontology”, “semantic predications”, and “merged” settings. The reason behind this is that Node2vec can well balance DFS and BFS in generating random walks to better work on different-structured graphs, and can thus learn high-quality node embeddings in different-structured graphs in contrast to other embedding learning approaches that focus on specific-structured graphs. This observation also justifies our COS design that employs random walks based on Node2vec to generate MeSH term sequences. Furthermore, the merged embedding outperforms respective embeddings from single data sources, showing that merging the three data sources is helpful in learning the MeSH term embedding.

Moreover, the most important finding in Tables 4–7 is that COS outperforms all the baselines on all datasets and all metrics. The finding answers the research question in the introduction that merging the three data sources will improve the embedding quality. The result also shows the effectiveness of COS, i.e., COS can effectively learn the MeSH term embeddings based on the corpus, ontology, and semantic predications data sources. It is also worth mentioning that COS embeddings outperform the merged embeddings with significance, justifying the advantage of COS over simply merging the embedding for different data sources.

#### Ablation study

In COS, we propose a sampling algorithm, ensuring that the numbers of sequences from different data sources are identical. To justify the sampling algorithm’s advantage, we conduct experiments comparing COS with sampling (up sampling) and COS without sampling (no sampling) on the four datasets. Moreover, we also compare COS sampling up and sampling down to the original number of sequences (up&down sampling), as described in the sequence sampling and merging subsection, to check whether the improvement comes from up sampling itself or from a greater number of sequences.

To mitigate the impact of random initialization, we run each setting ten times and present the averaged scores. Table 8 presents the results. We can observe from Table 8 that COS with sampling, both up&down sampling and up sampling, performs consistently better than COS without sampling in all datasets and on all metrics. The observation reveals that our proposed sampling algorithm can improve the quality of MeSH term embeddings in COS, i.e., it is beneficial to ensure that different data sources contribute equally to the MeSH term embedding learning. Moreover, we can observe from Table 8 that there is no clear difference between the performance of COS with up sampling and COS with up&down sampling, showing that the improvement in performance comes from the up sampling algorithm, or ensuring equal contributions from different data sources, rather than a greater number of sequences.

**Table 8:** The edge prediction results of COS using different sampling strategies.

Relation	Sampling	P(%)	R(%)	F1(%)	MAP(%)	AUROC(%)	AUPRC(%)
treat	no	90.07	90.07	90.07	86.19	90.07	92.61
	up&down	90.44*	90.44*	90.44*	87.04*	90.44*	93.06*
	up	91.38* †	91.38* †	91.37* †	88.30* †	91.38* †	93.78* †
interact	no	89.68	89.68	89.68	85.45	89.68	92.21
	up&down	90.35*	90.35*	90.34*	86.71*	90.35*	92.89*
	up	90.13*	90.13*	90.13*	86.34*	90.13*	92.69*
cause	no	89.17	89.17	89.17	84.98	89.17	91.92
	up&down	90.20*	90.20*	90.20*	86.66*	90.20*	92.85*
	up	90.09*	90.09*	90.08*	86.55*	90.09*	92.79*
affect	no	90.42	90.42	90.41	86.63	90.42	92.86
	up&down	91.36*	91.36*	91.36*	88.09*	91.36*	93.67*
	up	91.82* †	91.82* †	91.82* †	88.51*	91.82* †	93.92*

* denotes statistically significant difference (*p* < 0.001) compared to no sampling.

† denotes up’s statistically significant difference (*p* < 0.001) compared to up&down.

In another ablation study, to justify the generalizability of COS (i.e., COS is not tailored for SemMedDB), we exclude SemMedDB in learning the MeSH term embeddings and measure the performance of COS. Specifically, we compare COS with the three data sources (C&O&S) and COS with only the corpus and the ontology (C&O) data sources in learning the MeSH term embeddings. Table 9 presents COS with C&O and COS with C&O&S as the data sources. We can observe from Table 9 that COS with C&O&S always outperforms COS with only C&O with statistical significance. The observation once again justifies that using more data sources will improve the MeSH term embeddings’ quality. Besides, COS with C&O as the data sources also outperforms baseline embeddings with a single data source as in Tables 4–7. The finding indicates that the COS framework can generalize to data not present in the training data sources, showing its generalizability.

**Table 9:** The edge prediction results of COS and COS without using the semantic predications data source.

Relation	Data Source	P(%)	R(%)	F1(%)	MAP(%)	AUROC(%)	AUPRC(%)
treat	C&O	90.37	90.37	90.37	86.45	90.37	92.76
	C&O&S	91.38*	91.38*	91.37*	88.30*	91.38*	93.78*
interact	C&O	88.93	88.93	88.92	84.44	88.93	91.63
	C&O&S	90.13*	90.13*	90.13*	86.34*	90.13*	92.69*
cause	C&O	88.79	88.79	88.78	84.44	88.79	91.61
	C&O&S	90.09*	90.09*	90.08*	86.55*	90.09*	92.79*
affect	C&O	90.59	90.59	90.59	86.59	90.59	92.86
	C&O&S	91.82*	91.82*	91.82*	88.51*	91.82*	93.92*

* denotes satistically significant difference (*p* < 0.001) compared to C&O.

## Conclusions and future directions

We present a novel MeSH term embedding learning approach COS that incorporates the corpus, ontology, and semantic predications information of MeSH terms, which is the first MeSH term embedding that merges all the three data sources to the best of our knowledge. Experiments show that COS outperforms: 1) baselines using different embedding techniques and data sources, and 2) non-embedding-based baselines. The results empirically demonstrate the benefit of introducing multiple data sources in learning to represent MeSH terms and the effectiveness of COS.

Future directions include using the three data sources’ temporal information (i.e., the data sources change over time, and we can model such temporal change into the embeddings) to improve the MeSH term embeddings’ quality further.
